# Size does matter: crocodile mothers react more to the voice of smaller offspring

**DOI:** 10.1038/srep15547

**Published:** 2015-10-23

**Authors:** T. Chabert, A. Colin, T. Aubin, V. Shacks, S. L. Bourquin, R. M. Elsey, J. G. Acosta, N. Mathevon

**Affiliations:** 1 Equipe Neuro-Ethologie Sensorielle, ENES/Neuro-PSI, CNRS UMR9197, Université de Lyon/Saint-Etienne, France; 2 Equipe Communications Acoustiques, Neuro-PSI, CNRS UMR9197, Université Paris-Sud, France; 3 Centre National de la Recherche Scientifique, UMR 9197, Orsay, France; 4 Okavango Crocodile Monitoring Programme, Maun, Botswana; 5 Louisiana Department of Wildlife and Fisheries, Rockefeller Wildlife Refuge, Grand Chenier, Louisiana, USA; 6 Fundo Pecuario Masaguaral, Francisco de Miranda, Estado de Guárico, Venezuela; 7 Department of Psychology, Hunter College, City University of New York, USA

## Abstract

Parental care is widespread in Archosaurs (birds, crocodilians, dinosaurs and pterosaurs), and this group provides a useful model for the evolution of parent-offspring interactions. While offspring signalling has been well-studied in birds, the modulation of parental care in crocodilians remains an open question. Here we show that acoustic communication has a key role in the dynamics of crocodilian’ mother-offspring relationships. We found embedded information about the emitter’s size in juvenile calls of several species, and experimentally demonstrated that Nile crocodile mothers breeding in the wild are less receptive to the calls of larger juveniles. Using synthetized sounds, we further showed that female’ reaction depends on call pitch, an important cue bearing size information. Changes in acoustic interactions may thus go with the break of maternal care as well as dispersal of juvenile crocodilians. This process could have characterized other archosaurs displaying rapid early growth such as dinosaurs and pterosaurs.

Crocodilians and birds are the modern representatives of archosaurs, a monophyletic group that also includes the extinct dinosaurs and pterosaurs^1^,^2^. There is currently a great interest in understanding the evolution of the archausorian lineage^3^ since this group has led to a diversity of species showing developed cognitive and social abilities comparable to mammals, such as social cooperation (in birds^4^, and crocodilians^5^) and vocal learning (in birds^6^,^7^). As crocodilians diverged from birds more than 240 million years ago^8^, they are of primary interest in the reconstruction of ancestral archosaurian biology^9^. Together with genetic studies^10^,^11^, investigations on other biological traits of crocodilians will help in providing a comprehensive view of the archosaur evolution^12^,^13^,^14^. Specifically, knowledge about crocodilian social behaviours is an important issue, which could bring relevant information about the process of their implementation during the history of archosaurs. Here we focus on parental care, a behavioural trait widely shared among archosaurs.

Archosaurs are oviparous, and one or both parents of all but a few species take care of the eggs by incubating and/or guarding the nest, and the hatchlings by feeding and/or guarding them (reviewed for birds^15^; for crocodilians^2^,^16^,^17^). Moreover, there are substantial records showing that fossil archosaurs also displayed parental care^18^,^19^,^20^,^21^ (although the case for post-hatching care in pterosaurs is rather weak^22^). Thus, parental care constitutes a behavioural feature that deserves to be studied in living archosaurs with the aim of understanding the phylogenetical, physiological and ecological factors that have driven their evolution. Specifically, deciphering how interactions within the family are modulated by communication signals represents a major focal point.

Similarly to what is observed in birds, the acoustic communication channel is used by crocodilians in the context of parent-offspring interactions^17^,^23^,^24^,^25^,^26^. However, while numerous studies have deciphered how acoustic signals mediate kin interactions in birds^27^, our knowledge concerning crocodilians remains scant^28^. Experimental investigations recently demonstrated that juvenile calls carry relevant information for both siblings and the mother. Thus, females guarding their nest react to the calls of near-term embryos by providing assistance during the hatching process^17^,^29^. Later, vocalizations continue to mediate mother-juvenile interactions: when young are seized by a predator, they emit “distress” calls that attract the mother^25^,^30^. Similarly, newborn vocalizations mediate hatching coordination^29^ and later, group cohesion^30^.

After several weeks or months^17^, the family group splits: the mother stops guarding the juveniles, even actively chasing them away^31^, and the juveniles disperse. The reasons for this shift in both maternal and juvenile behaviours are not known. It is likely that some ecological factors are involved, with e.g. a decrease of predation pressure as juveniles grow larger, together with an increase of competition for food and antagonistic behaviour between siblings^31^,^32^,^33^. Maternal interaction may decrease due to hormonal modifications^34^. Acoustic signals that mediate mother-young interactions might also become less efficient in eliciting behavioural responses. Thus, it has been demonstrated that the acoustic structure of young Nile crocodiles *Crocodylus niloticus* calls changes from the first to the fourth day after hatching, with a progressive decrease in the fundamental frequency, *i.e.* the pitch of the call^35^. Though no study has yet investigated the acoustic modifications of calls further induced by the juvenile’s growth, it is likely that they are substantial and that they might provide information modulating the mother’s behaviour. Thus, it is not known if crocodile mothers could be more likely to provide protection in response to calls from smaller juveniles than to calls of larger ones.

The aim of the present study is to characterize the growth-induced modifications of juvenile vocalizations in crocodilians, and to investigate the potential effect of these changes on maternal response. We recorded individuals of several species belonging to the two main families Alligatoridae and Crocodylidae, and assessed the effect of body size on the call structure. Using playback experiments with both natural and synthetic signals, we further tested the reaction of Nile crocodile females to calls from juveniles of different sizes. Here we present the first experimental evidence that size-related information embedded in the calls of juvenile crocodilians modulates maternal care.

## Results

### Juvenile calls vary with the emitter’s body size

**Figure 1:**
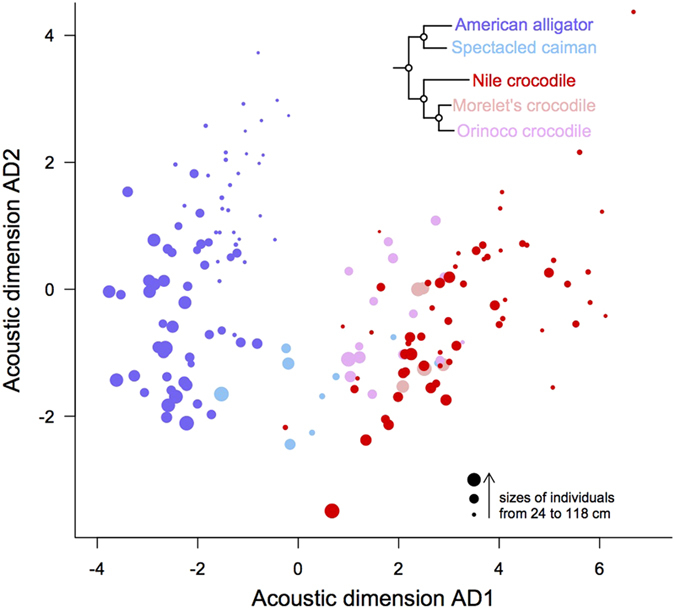
Distribution of crocodilian juvenile calls within the acoustic space. Each circle represents a single individual. The diameter of disks is proportional to the individual’s body length. Alligatoridae are in dark blue (American alligator) and sky blue (spectacled caiman). Crocodylidae are in red (Nile crocodile), plum (Morelet’s crocodile), and pink (Orinoco crocodile). The acoustic structure of calls was first described using 13 parameters in the frequency and temporal domains (see text for a description of the parameters), further reduced using a principal component analysis into two independent Acoustic Dimensions (AD1 and AD2). We then calculated the mean AD1 and AD2 for each recorded individual, so that each individual could be positioned on the two-dimensional acoustic space. The first axis of the acoustic space (AD1) is mainly related to the distribution of energy among the frequency spectrum (with higher scores meaning wider frequency band). The second axis of the acoustic space (AD2) is mainly related to the pitch (with higher scores indicating higher pitched calls). The first axis of the acoustic space separates out well the American alligator (family Alligatoridae) from the Crocodylidae. Both axes contribute to separate out juveniles based on their individual body length (see text for details). The tree shows phylogenetical relationships between species^8^,^10^,^59^.

Calls of the following species were recorded and analyzed: American alligator *Alligator mississipiensis*, Nile crocodile *Crocodylus niloticus*, spectacled caiman *Caiman crocodilus*, Morelet’s crocodile *Crocodylus moreletii*, and Orinoco crocodile *Crocodylus intermedius*. Calls were emitted spontaneously when animals were hand captured. The calls’ acoustic structure was characterized using a set of 13 temporal and spectral parameters describing both the distribution of energy among the frequency spectrum and the call pitch (see Methods). To test for the presence of size-related information in the calls, a multivariate principal component analysis was performed to reduce these 13 non-independent acoustic parameters into two independent Acoustic Dimensions (AD1 and AD2), and linear mixed effect models (LME) were used with AD1 and AD2 as dependent measures (fixed effects: individual size, crocodilian species; random effect: individual identity). The acoustic structure of juvenile calls depended on the size of recorded individuals (Fig. 1; LME on AD1 scores with individual size as a fixed effect: χ^2^ = 52.2, df = 1, *P* < 0.0001; AD2 scores: χ^2^ = 83.60, df = 1, *P* < 0.0001). The acoustic factors that loaded the most on AD1 were parameters describing the distribution of energy among the frequency spectrum (Table 1). Calls of larger juveniles showed more energy in the lower part of their frequency spectrum than calls from smaller individuals. For instance, the mean frequency of Nile crocodile calls decreased from 2.5 to 2.1 kHz for juveniles measuring from 20–40 cm total length (TL) to 80–100 cm TL (Table 2). The second acoustic dimension AD2 was mainly explained by the call pitch (Table 1), with calls from smaller individuals being higher pitched (Fig. 1). For instance, the maximum pitch for 20–40 cm Nile crocodiles reached 969 Hz (with a mean around 640 Hz), while it reached only 529 Hz (with a mean around 440 Hz) for 80–120 cm individuals.

**Figure 2:**
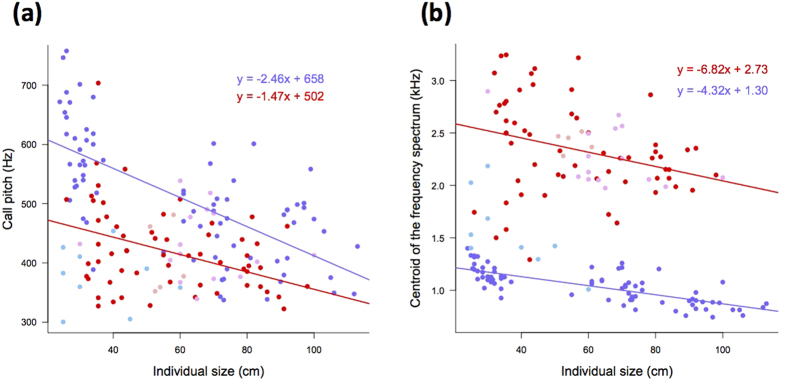
Correlations between individual size (total body length) and (a) the calls’ mean pitch, and (b) the calls’ centroid of the frequency spectrum. Each dot represents a single individual. Both acoustic parameters decrease with individual size, underlying that smaller individuals utter higher pitched calls with a wider frequency bandwidth^59^. Alligatoridae are in dark blue (American alligator) and sky blue (spectacled caiman). Crocodylidae are in red (Nile crocodile), plum (Morelet’s crocodile), and pink (Orinoco crocodile). Regression lines are shown for the alligator and the Nile crocodile only. Slopes of the regression lines are not significantly different between both species (see results).

In addition to demonstrating the importance of individual size as a factor explaining call structure, the comparison between juvenile calls underlined differences between crocodilian species (Fig. 1; LME on AD1 scores with species identity as a fixed effect: χ^2^ = 322, df = 4, *P* < 0.0001; AD2 scores: χ^2^ = 61.6, df = 4, *P* < 0.0001). However, we found no significant effect of the interaction between species and individual size (LME on AD1 scores with interaction between species identity and individual size as a fixed effect: χ^2^ = 4.45, df = 4, *P* = 0.348; AD2 scores: χ^2^ = 4.63, df = 4, *P* = 0.327). We calculated, separately for the American alligator and the Nile crocodile (two species well-represented in our data set and representative respectively of the Alligatoridae and the Crocodylidae lineages), linear regressions between individual size and both the call pitch and the frequency spectrum centroid (Fig. 2a,b). Although the intercepts of both regression lines were different between both species (for the mean pitch: *t* = −4.66, *P* < 0.0001; for the centroid: *t* = 10.20, *P* < 0.0001), their slopes did not differ significantly (for the mean pitch: *t* = 1.86, *P* = 0.0645; for the centroid: *t* = −1.13, *P* = 0.262). Both the American alligator and the Nile crocodile–and probably all crocodilians- are thus likely to follow a similar general rule for coding information about body size in their juvenile calls.

### Calls of small juveniles are more attractive to nesting females in the wild

**Figure 3:**
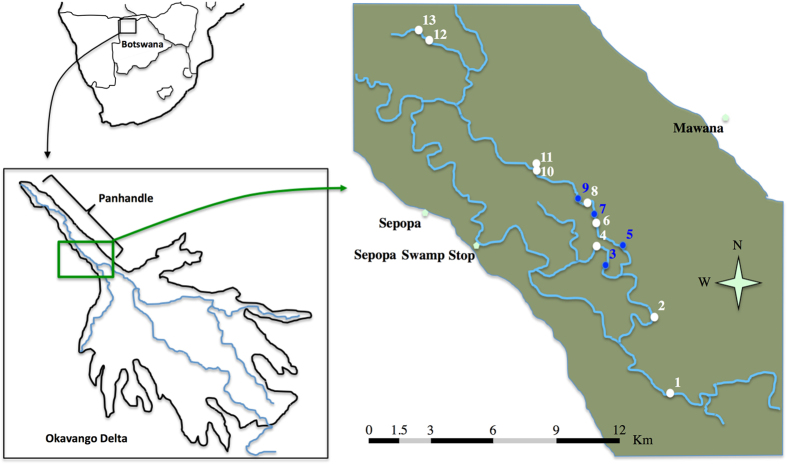
Map of the study area in the Panhandle, Okavango Delta, Botswana. Numbered dots indicate the position of the 13 crocodile nests mapped in December 2014. White dots correspond to the nine nests where we did the playback experiments in January 2015.

**Figure 4:**
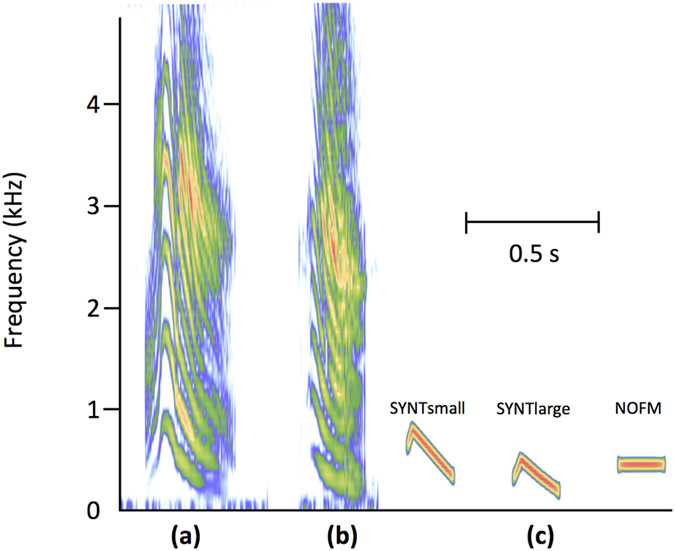
Spectrograms of experimental calls. (**a**) Call emitted by a Nile crocodile juvenile measuring 35.5 cm (total body length; “small” individual). (**b**) Call emitted by a 64.5 cm Nile crocodile juvenile (“large” individual). (**c**) Synthetic signals; from left to right: high-pitched synthetic pure tone modulated in frequency (SYNTsmall); low-pitched pure tone modulated in frequency (SYNTlarge); unmodulated pure tone (NOFM, control signal). Graphical representation produced with the R package Seewave (Sueur *et al.* 2008) with spectrograms set to Hanning window and a FFT window length of 512 with 90% overlap.

**Figure 5:**
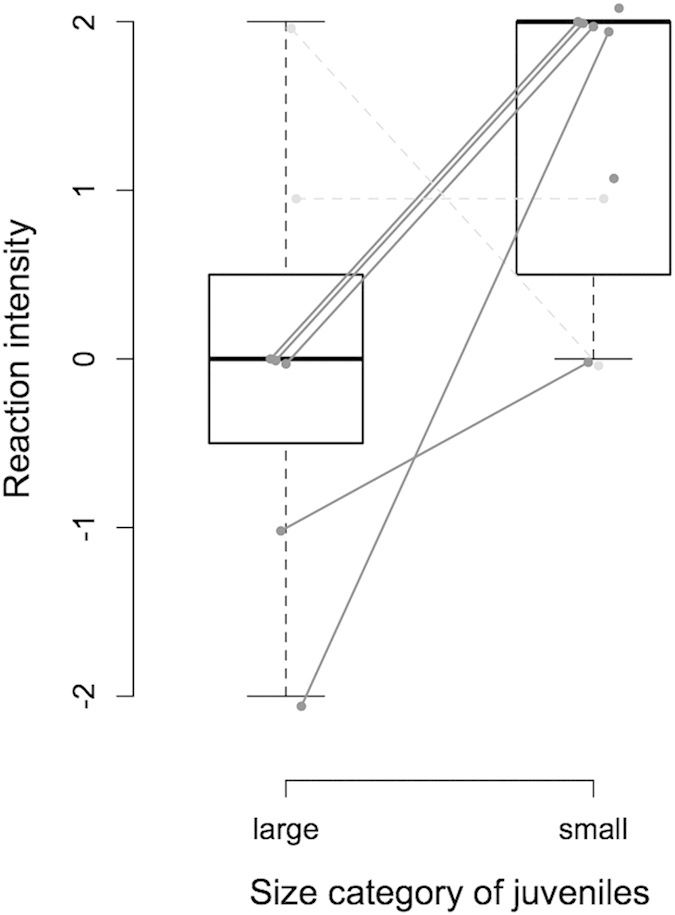
Behavioural response of wild breeding female Nile crocodiles to calls of “large” and “small” juveniles. Most females have been tested with both stimuli (the lines on the figure link responses from the same females; those females which have been tested once are represented by single dots). Solid grey lines = females that approached more towards the loudspeaker when challenged with calls from a small juvenile than when tested with calls from a large juvenile. Dashed grey lines = females that responded equally to both experimental signals or approached more towards the loudspeaker when hearing calls from a large juvenile. Positive values on the reaction intensity y-axis mean an approach to the loudspeaker. Negative values mean a retreat (see text for details).

To test if the size-related information embedded in juvenile calls was of significant importance to mothers, female Nile crocodiles breeding in the wild (Okavango Delta, Botswana, Fig. 3) were challenged by playing calls emitted by small (hatchling, total body length 28–36 cm) and large (63.5–98 cm) juveniles (Fig. 4a,b, Supplementary Audio 1). The tested females reacted differently depending on the call’s size category (Fig. 5; GLM: χ^2^ = 5.53, df = 1, *P* = 0.019, *N* = 9). In response to calls from hatchlings, females moved more towards the loudspeaker: seven individuals out of the nine tested approached, with five coming to within one meter, and only two females did not react to these calls. Conversely, calls from large juveniles hardly elicited an approach: three females out of the seven tested did not move, and two individuals even retreated. Only one female approached closer to the loudspeaker in response to the calls of a large juvenile than to the calls of a hatchling.

### Variation of call pitch modulates female response

Based on the results of the acoustic analysis, we tested if the pitch of juvenile calls, an important cue bearing size information, could explain by itself the variation in females’ reaction. A previous study showed that female (mature adults) Nile crocodiles were reactive to a simplified synthetic copy of newborn’s calls where all harmonics except one were removed (“pure” sound), provided that the frequency modulation of the sound was kept identical to the original call^36^. On the basis of this result, female Nile crocodiles were tested using three synthetic experimental signals (Fig. 4c, Supplementary Audio 1): (1) a signal (SYNTsmall) constiting of a pure tone modulated in frequency (mimicking a call with only the first harmonic with its natural frequency modulation) with a pitch within the range of the calls from hatchlings, (2) a signal (SYNTlarge) identical to SYNTsmall except that its pitch was within the range of the calls from large juveniles, (3) a control signal constituted by a pure tone not modulated in frequency (NOFM).

**Figure 6:**
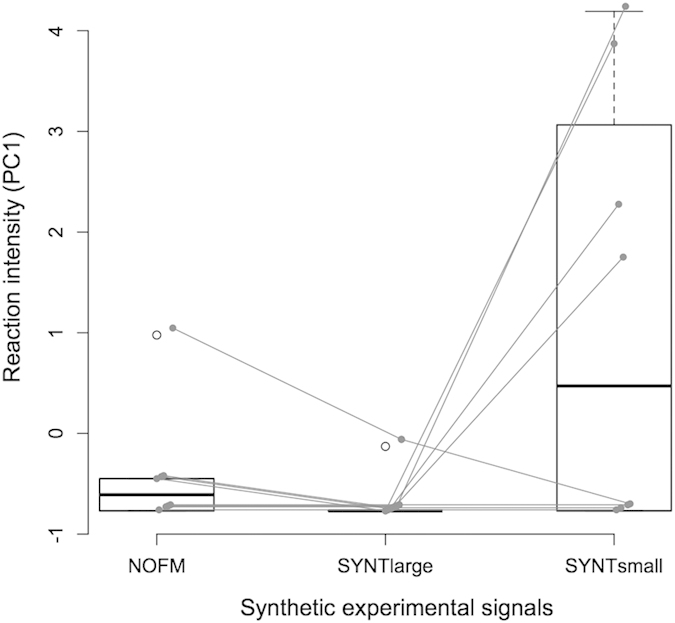
Behavioural response of clusters of captive female Nile crocodiles to experimental synthetic signals. The responses are expressed by composite scores (PC1) that integrate the assessment of several behaviors (see text for details). Higher scores mean more females reacting and closer approach towards the loudspeaker. Dots represent each cluster’s response. Grey lines link responses from the same cluster. NOFM = pure tone at constant frequency of 500 Hz. SYNTlarge = pure tone modulated in frequency with a pitch in the range of calls from large juveniles. SYNTsmall = pure tone modulated in frequency with a pitch in the range of calls from small juveniles.

As the experiments were conducted in a zoo, it was impossible to test animals individually. Experimental signals were thus played to groups of 4–20 adult females (see Methods). We first checked if these captive females exhibited a differential response to calls from small and large juveniles in line with that observed with wild free-ranging crocodiles in the Okavango. The number of females approaching the loudspeaker was indeed significantly higher in response to calls recorded from small individuals than to calls recorded from large ones (GLM: χ^2^ = 5.10, df = 1, *P* = 0.024, *N* = 7): calls from small individuals attracted between one and 10 females during six out of the seven experiments while calls from large individuals induced the venue of only one to two females during three out of the seven experiments (none approached on the other four trials). Eight clusters of individuals were then challenged with the three different synthetic stimuli. The reaction of females depended on the experimental signal (Fig. 6; GLM: χ^2^ = 0.02, df = 2, *P* = 0.010). Adult female Nile crocodiles were more attracted by the high pitched synthetic signal SYNTsmall than by the control pure tone NOFM (multiple comparison test: Z = −3.053, *P* = 0.0064, *N* = 8). They also responded more to the high-pitched SYNTsmall than to the lower-pitched signal SYNTlarge (multiple comparison test: Z = −2.59, *P* = 0.026, *N* = 8). Conversely, there was no significant difference in the behaviour of females tested by the control pure tone and by the lower-pitched signal (multiple comparison test: Z = −0.74, *P* = 0.739, *N* = 8).

## Discussion

The acoustic analysis shows that vocalizations emitted by juvenile crocodilians contain reliable information about the emitter’s size, with smaller individuals uttering higher pitched calls with a wider frequency bandwidth. Furthermore, playback experiments on Nile crocodiles in the wild demonstrate that this information is accessible to adult breeding females, which approached the loudspeaker more when hearing calls of smaller individuals. Finally, the experiments in captivity demonstrate that the call pitch, which gets lower when juveniles grow larger, modulates the female’s behaviour.

The influence of growth on vocalizations has been shown in many animal species^37^, and is linked to the growth-induced modifications of the vocal vibratory membranes and the vocal tract (e.g. larger folds produce lower fundamental frequency and longer tracts allow lower resonance frequencies in mammals^38^). Although crocodiles do not have a specialized vocal organ as birds and mammals do, they have a vibratory membrane–the palatal valve- which is used for sound production^28^,^39^,^40^. This valve grows with the animal, as do the resonators constituted by the nasal and buccal cavities, and this could explain modifications of the call’s acoustic properties, respectively the pitch and the distribution of energy among the frequency spectrum^38^.

Our analysis recorded significant differences between the juvenile calls of Alligatoridae and Crocodylidae. This finding confirms previous studies demonstrating inter-specific differences in the acoustic structure of calls^36^. However, this also suggests that call structure similarities between species correspond to phylogenetical proximity: the position of the Nile crocodiles’, the Orinoco crocodiles’, and the Morelet’s crocodiles’ calls in the acoustic space reflects their phylogenetical link^41^. Although a larger sample size would be necessary to draw specific conclusions, juvenile calls might thus be used as phylogenetic markers among crocodilians. However, and more importantly for the purpose of our study, the call analysis underlines that all species studied follow a similar acoustic allometric rule, with the pitch constituting a major parameter coding for body size.

Results of the playback experiments indicate that adult breeding females were less likely to come to the loudspeaker when calls of larger juveniles were emitted. This result suggests that crocodile mothers pay selective attention to calls emitted by smaller individuals (hatchlings). This behaviour could suggest maternal vigilance is increased relative to offspring highly susceptible to predation^17^. It is known from observations in the wild that Nile crocodile mothers abandon their young after a few weeks^42^,^43^,^44^, and that young enter a dispersal phase at approximately 1.2 m length; probably because of ontogenetic shift in diet^45^. Although a number of factors may be involved in the female’s decision to leave her offspring^45^, less efficient acoustic solicitation from the young could weaken the mother-offspring link.

In the wild we have tested both females still attending the nest and females attending hatchlings. Although we did not document any evidence of difference in their reaction towards our played back signals, the small sample size does not allow for definitive conclusions. It would be interesting to see if a female whose offspring have already hatched would be more likely to respond to distress calls than a female whose eggs are still incubating.

Our playback experiments with synthetic signals were performed in captivity. We thus were unable to test isolated mothers attending their young, but instead played back experimental signals to groups of captive females where it was impossible to know how many of them had eggs at the time. These results should thus be interpreted cautiously. However, given the preferential attentiveness of captive females towards calls of smaller individuals, we assume that the results obtained with synthetic signals bring significant information about the importance of call pitch as a feature modulating female’s reaction.

In this study, we focused on acoustic variations linked to the animal’s size. There is obviously a link between size and age (e.g. crocodiles which are 10 years old are generally expected to be around 2 m long in the Okavango Delta, Shacks *pers.obs.*). However, growth in crocodilians is highly dependent on external factors such as feeding success and temperature. During the first year of life, the crocodile’s size can vary greatly. As susceptibility to predation depends on size more than on age^17^, information directed to a mother regarding the offspring’s size seems particularly relevant.

In conclusion, our study shows that vocalizations of juvenile crocodilians bring reliable information that is of importance to the mother. These sound signals may intervene in the dispersal of the crocodile family by modulating maternal reaction. This result adds a new piece of information on the importance of vocal communication signals during mother-offspring relationships in crocodiles.

From a broader point of view, the present work underlines the importance of body size-related information in signals addressed by offspring to their parents. It is well-known from bird and mammal studies that parents care about “dynamic” (e.g. satiety state) as well as “static” (e.g. individual identity) information encoded in offspring signals^46^. Additionally, it has been demonstrated by a number of investigations that information about body size is of tremendous importance in adults, e.g. when competing for mates^47^,^48^. From the best of our knowledge, the present study brings the first experimental demonstration that parental care can be modulated by offspring signalling body size-related information through their vocalizations.

Finally, in spite of recent advances in our understanding of the behavioural traits of ancient archosaurs such as dinosaurs, it still remains a challenge to obtain reliable information about their social interactions and particularly parental care. The present research could thus be of interest beyond the field of crocodilian biology by suggesting that size-related information in juvenile’s acoustic signal could have been of importance in other non-crocodilian archosaurs found to provide post-hatching parental care. Specifically, rapid early growth has been demonstrated in dinosaurs^49^,^50^,^51^,^52^ and pterosaurs^53^. Additionally, parental care appears as a general shared feature in these groups and acoustic communication was certainly widely used^19^,^20^. Although more data–e.g. in birds- are clearly required to support this hypothesis, we suggest that modulation of parental care by juvenile vocalizations as demonstrated here with crocodilians could be rooted deeply in the archosaurian evolutionary tree.

## Methods

### Acoustic analysis of juvenile calls

The details of recorded individuals were as follows (see also Table 2): American alligator (*N* = 76, measuring 24 to 113 cm from the extremity of the snout to the end of the tail, individuals aged 2 months to approximately 3 years old, recordings location: Rockefeller Wildlife Refuge, Louisiana, USA), Nile crocodile (*N* = 60, 26–118 cm, 1 week to 2 years, Parc Djerba Explore, Tunisia; La Ferme aux Crocodiles, Pierrelatte, France; Okavango Delta, Botswana, Africa), spectacled caiman (*N* = 9, 25–60 cm, aged 2 weeks to 1 year, Hato Masaguaral, Venezuela), Morelet’s crocodile (*N* = 5, 51–61 cm individuals aged 10 months, La Ferme aux Crocodiles, Pierrelatte, France), Orinoco crocodile (*N* = 14, 30–100 cm individuals aged 4–7 months, Hato Masaguaral, Venezuela). Calls were recorded using a SCHOEPS MK4 cardioid microphone, positioned at 20 cm from the animal’s snout, and connected to a NAGRA-LB recorder. For the analysis purpose, we selected between 1 and 20 calls/individual (mean = 15.0 ± 5.0) from the recordings, depending on suitable acoustic quality.

Juvenile calls are signals with a complex acoustic structure displaying a fundamental frequency and a series of harmonics, modulated in frequency^28^. We performed acoustic analyses using Seewave R package^54^ and PRAAT software^55^. The first part of the analysis characterized the distribution of energy within the call frequency spectrum. We extracted the frequency spectrum under Seewave (FFT window = 1024; overlap = 99%), and calculated the following spectrum properties within a 0–5 kHz bandwidth: the mode (maximal frequency) of the frequency spectrum, the first quartile of energy (Q25), i.e. the frequency value corresponding to 25% of the total energy spectrum, the third quartile of energy (Q75), i.e. the frequency value corresponding to 75% of the total energy spectrum, the interquartile range (IQR), i.e. the difference between Q75 and Q25, the centroid of the frequency spectrum (cent), the skewness, a measure of spectrum asymetry, the kurtosis, a measure of spectrum peakedness, the spectral flatness (sfm), i.e. the ratio between the geometric mean and the arithmetic mean of the spectrum (this ratio can vary between 0 and 1, with sfm of a noisy sound tending towards 1, and sfm of a pure tone signal tending towards 0).

The second part of the analysis characterized the pitch (fundamental frequency or F0) and the intonation (F0 contour variation) of the calls. The F0 contour was extracted under PRAAT. We systematically inspected the extracted pitch contour and verified it using a narrow band spectrogram displaying the first 0–2000 Hz of the signal. Spurious octave jumps were manually corrected by selecting the appropriate F0 candidate values. Each extracted F0 contour was used to derive the following parameters: the F0 value at the beginning of the call (start pitch), the maximum and minimum F0 values (max pitch and min pitch), the F0 mean value (mean pitch), the F0 value at the end of the call (end pitch).

To test for the presence of size-related information in the calls, we first performed a multivariate principal component analysis to reduce the 13 non-independent acoustic parameters described above into two independent Acoustic Dimensions (AD1 and AD2). We further used linear mixed effect models with AD1 and AD2 as dependent measures (fixed effects: individual size, crocodilian species, and interaction between size and species; random effect: individual identity; package lme4, R version 3.1.2). P values were obtained with likelihood-ratio tests comparing the fit of full models with reduced models lacking each fixed effect. Although the smallest individual was also the youngest, and the longest presumably the oldest, we assumed that age is secondary compared to size as a potential factor determining the acoustic structure of juvenile calls. Thus, the size of one-year Nile crocodiles spanned from 32 to 92 cm, *i.e.* 65% of the total size range (26–118 cm) of the recorded individuals. We thus omitted the age in the analysis.

### Playback experiments 1: Assessing maternal response in the wild

The experiments took place in the delta of the Okavango River in Botswana, Southern Africa (Fig. 3). During the dry season, breeding females are found in the main river channel known as the “Panhandle” region of the Okavango Delta^56^,^57^. In December 2014, the locations of 13 nests were plotted in the area from Sepopa to Seronga, each nest being attended by an adult female (Fig. 3). Playbacks were conducted between the 10^th^ and 20^th^ of January 2015, which corresponds with the peak hatching period in the Panhandle. At the time of experiments, nine females were still attending their nest (n = 6) or had hatchlings (n = 3), while four nests were abandoned, mostly due to fires and predation by the water leguaan *Varanus niloticus*.

#### Acoustic stimuli

To test whether breeding female crocodiles react differently to calls depending on juvenile’s size, playback experiments were performed with calls of juveniles belonging to two different size categories (Fig. 4a,b): “small” individuals (measuring 28–36 cm) and “large” individuals (measuring between 63.5 and 98 cm).

#### Playback tests

Eight out of the nine females were tested at night, when it was easier to locate crocodiles present in the river (the eyes reflecting the light of spotlights). Prior to playback, the position of the female and of the nest were assessed and a remote-controlled amplified loudspeaker (FoxPro Fury©) was attached to papyrus on the river bank near the water, close to the nest. The observers remained in a boat at 30–50 meters from the loudspeaker and the female. One female was tested during the day, from land, since her nest was inaccessible by boat. In this case, we positioned the loudspeaker on the water edge, near the nest, and a single observer remained 15 meters away hidden by vegetation. All females were tested while they were in the water, at 10–30 meters from the nest and thus from the loudspeaker. After 10–30 minutes of silence, we played the experimental signals.

Seven females were tested during two playback sessions (one session with calls from a small juvenile, the other with calls from a large juvenile, in a balanced order between females), separated by at least two days. Two females received a single playback session (with calls from a small juvenile) because they were gone before we were able to test them again. A playback session was constituted by a maximum of two consecutive series of juvenile calls recorded from a single individual, either small or large (39 ± 4 calls/series; series duration = 1 minute; 1 minute of silence between both series). If a behavioural reaction was observed during the first series of calls, the experimenters did not broadcast the second series to limit habituation. Each female was tested with calls from different juveniles, thus avoiding pseudoreplication. Calls were played back at a natural intensity (intensity level: 63 ± 5 dB_SPL_ at 1 m from the loudspeaker).

#### Assessment of females’ behavioural reaction to experimental signals

Females responded to playback either by approaching the loudspeaker or by retreating. The approach could be strong, with an immediate approach to within one meter from the loudspeaker, or less intense by swimming for only few meters (no more than 5 m) in the direction of the loudspeaker. Females that retreated did so for a few meters (no more than 5 m) or on a larger distance. Some of the playbacks were followed by an apparent absence of reactiveness, the female staying at the place she was before the broadcast of the sound stimuli. The following ethological intensity scale was used to quantify the females’ responses: +2 = approach towards the loudspeaker (more than half the distance between the loudspeaker and the female’s initial position); +1 = partial approach (less than half the distance between the loudspeaker and the female’s initial position); 0 = no response; −1 = small retreat (less than 5 meters); −2 = strong retreat (more than 5 meters). To test for a significant effect of the category of stimulus (“small” juvenile versus “large” juvenile), a linear mixed effect model was used with the reaction intensity as the dependent measure (fixed effect: category of stimulus–*i.e.* calls from “small” or “large” individuals; random effects: nest identity, playback order). The P value was obtained with a likelihood-ratio test comparing the fit of the full model with a reduced model lacking the fixed effect.

### Playback experiments 2: Does the pitch of juvenile calls modulate females’ reaction?

These experiments were performed on captive females at the zoo “La Ferme aux Crocodiles” (housing 350 adult Nile crocodiles in a 8000 m^2^ tropical greenhouse; located in Pierrelatte, France). Although it was possible to approach animals, it was impossible to test them individually. Experimental signals were thus played back from 7–8 locations where clusters of 4–20 adult females were present (closest individual at least at 5 m from the loudspeaker; intensity level: 61 ± 4 dB_SPL_ at 1 m from the loudspeaker). These locations were as far away as possible to each other (more than 30 m apart). The experiments took place in spring and late summer of 2014, a period where mothers should normally be with their nest or hatchlings (at La Ferme aux Crocodiles, hatching occurs from late June to the beginning of September; eggs are removed from nests before hatching but mothers still display the typical nest guarding behaviour^29^). At this period, females usually remain in the same area for days, so it is likely that the composition of clusters remained stable during our experiments.

Seven clusters of females were first tested with calls from juveniles belonging to two different size categories, “small” and “large”, as during the experiments in the Okavango. The 7 clusters were tested with both types of stimuli, on two consecutive days. Broadcast sequences contained 12–25 calls (silence interval between calls = 0.8 ± 0.2 s; sequence duration = 25 s). To avoid pseudo-replication, the clusters were exposed to calls recorded from different individuals. The order of both stimuli was balanced between clusters. Sounds were played through a loudspeaker positioned on the shore of the basins. A simple measurement of behavioural response was used, by counting how many females approached towards the loudspeaker during the playback and one minute after the playback (total duration of the observation period = 1 min 25 s). To test for a significant effect of the category of stimulus, a linear mixed effect model was performed with the number of females approaching the loudspeaker as the dependent measure (fixed effect: category of stimulus–*i.e.* calls from “small” or “large” individuals; random effects: cluster location, playback order). The P value was obtained with a likelihood-ratio test comparing the fit of the full model with a reduced model lacking the fixed effect.

For the experiments with synthetic signals, the SYNTsmall signal had a pitch in the range of the calls from small juveniles (start pitch = 630 Hz; mean pitch = 638 Hz; end pitch = 350 Hz; sound duration = 160 ms). The SYNTlarge was in the range of the calls from large juveniles (start pitch = 300 Hz; mean pitch = 384 Hz; end pitch = 200 Hz; sound duration = 160 ms). The pure tone NOFM was fixed at a constant pitch of 500 Hz (no frequency modulation; same duration as the two other synthetic sounds). Each cluster of females was successively tested with a series of these three synthetic signals in a balanced order. A playback series was constituted by eight repetitions of the same synthetic sound, separated by two seconds of silence. The loudspeaker (Megavox Pro©) was placed on the shore of the basins. Once the loudspeaker was positioned on the bank, we waited at least 5 minutes before initiating the experimental signals.

The behavioural response of adult females within a cluster was assessed using three criteria:

1. orientation (0 = none of the females orientated towards the loudspeaker; 1 = at least one female turned her head in the direction of the loudspeaker; 2 = at least one female orientated her whole body in the direction of the loudspeaker);
2. number of females approaching the loudspeaker during the playback;
3. maximal approach realized by at least one female from the cluster (0 = no approach; 1 = less than half the distance between the loudspeaker and the cluster of females; 2 = more than half the distance between the loudspeaker and the cluster of females).

Instead of separately analyzing these three non-independent measures of behavioural response, they were collapsed into a composite score using a principal component analysis (PCA)^58^. The first principal component (PC1) was chosen as a unique composite score for behavioural response as it fairly represents the strength of the response to playback (with higher scores meaning more females reacting and closer approach to the loudspeaker). To test for the effect of the played back signals on the females’ behavioural reaction, we used a linear mixed effect model with PC1 as the dependent measure (fixed effect: category of stimulus *i.e.* calls from “small” or “large” individuals; random effects: cluster location, playback order). The P value was obtained with a likelihood-ratio test comparing the fit of the full model with a reduced model lacking the fixed effect. To compare between types of experimental signals, this analysis was followed by post-hoc multiple comparison tests (function glht in multcomp R package).

**Ethical note.** All experimental protocol in the Okavango were approved by the Ministry of Environment, Wildlife and Tourism of Botswana (permit no EWT 8/36/4 XXVI). All experimental protocols in captivity were approved by the Institutional Animal Ethical Committee of the University of Saint-Etienne (Authorization no 42–218-0901SV09). After the experiments, the animals were monitored to assess potential deleterious effects of our investigations. Juveniles recorded in captivity then followed a normal growth pattern, without any mortality. Females in the wild did not prematurely abandon their nest or hatchlings.

All experiments were performed in accordance with the approved guidelines and regulations.

## Additional Information

**How to cite this article**: Chabert, T. *et al.* Size does matter: crocodile mothers react more to the voice of smaller offspring. *Sci. Rep.* **5**, 15547; doi: 10.1038/srep15547 (2015).

## Supplementary Material

Supplementary Audio legend

Supplementary Audio 1

## Figures and Tables

**Table 1:** Factor loadings on the two acoustic dimensions calculated from the acoustic parameters describing juvenile calls.

Acoustic parameters	Acoustic Dimension AD1 (% of variance = 55.4%)	Acoustic Dimension AD2 (% of variance = 19.1%)
mean pitch (Hz)	−0.188	**0.946**
start pitch (Hz)	−0.720	0.577
max pitch (Hz)	−0.702	0.597
min pitch (Hz)	0.607	0.591
end pitch (Hz)	0.603	0.574
maximal frequency (Hz)	**0.911**	0.025
Q25 (Hz)	**0.909**	0.041
Q75 (Hz)	**0.976**	0.013
IQR (Hz)	0.807	−0.029
cent (Hz)	**0.970**	0.011
skewness	−0.531	−0.316
kurtosis	−0.371	−0.337
sfm	0.898	−0.052

The first principal component is mainly related to the distribution of energy among the frequency spectrum. The second principal component is mainly related to the mean pitch of the calls.

**Table 2:** Acoustic characteristics (mean ± sd) of the calls of juvenile crocodilians in function of body size.

Number of recorded individuals (mean number of calls per individual, min-max)	Size category				
	20 – <40 cm	40 – <60 cm	60 – <80 cm	80 – <100 cm	100 – <120 cm
*American alligator* (19 calls/individual, 10–20)	31	1	23	15	6
*Spectacled caiman* (12 calls/individual, 5–20)	5	3	1	–	–
*Nile crocodile* (12 calls/individual, 3–20)	18	17	11	13	1
*Morelet’s crocodile* (19 calls/individual, 16–20)	–	4	1	–	–
*Orinoco crocodile* (7 calls/individual, 1–19)	1	1	10	1	1
mean pitch (Hz)					
* American alligator*	593 ± 83	418	461 ± 70	459 ± 73	405 ± 55
* Spectacled caiman*	376 ± 49	383 ± 75	358	–	–
* Nile crocodile*	456 ± 93	416 ± 58	412 ± 51	389 ± 47	222
* Morelet’s crocodile*	–	413 ± 67	377	–	–
* Orinoco crocodile*	432	405	433 ± 70	402	413
start pitch (Hz)					
* American alligator*	1100 ± 179	823	1128 ± 239	1092 ± 219	926 ± 200
* Spectacled caiman*	485 ± 98	665 ± 195	658	–	–
* Nile crocodile*	596 ± 135	480 ± 64	463 ± 67	375 ± 57	321
* Morelet’s crocodile*	–	732 ± 148	603	–	–
* Orinoco crocodile*	608	652	690 ± 172	476	770
max pitch (Hz)					
* American alligator*	1117 ± 183	893	1135 ± 233	1094 ± 217	929 ± 198
* Spectacled caiman*	553 ± 77	678 ± 192	658	–	–
* Nile crocodile*	635 ± 141	502 ± 65	508 ± 61	439 ± 49	351
* Morelet’s crocodile*	–	821 ± 226	671	–	–
* Orinoco crocodile*	623	657	730 ± 153	671	790
min pitch (Hz)					
* American alligator*	290 ± 36	147	161 ± 21	149 ± 30	144 ± 19
* Spectacled caiman*	187 ± 28	170 ± 15	179	–	–
* Nile crocodile*	316 ± 98	339 ± 68	308 ± 69	311 ± 49	133
* Morelet’s crocodile*	–	170 ± 15	176		
* Orinoco crocodile*	231	268	269 ± 40	221	231
end pitch (Hz)					
* American alligator*	292 ± 37	150	166 ± 19	153 ± 30	150 ± 21
* Spectacled caiman*	187 ± 28	171 ± 15	184	–	–
* Nile crocodile*	318 ± 102	343 ± 69	316 ± 73	326 ± 56	136
* Morelet’s crocodile*	–	172 ± 18	176	–	–
* Orinoco crocodile*	231	269	277 ± 40	244	239
maximal frequency (Hz)					
* American alligator*	785 ± 182	474	538 ± 63	475 ± 59	442 ± 43
* Spectacled caiman*	1401 ± 679	706 ± 126	511	–	–
* Nile crocodile*	2671 ± 1192	2648 ± 907	2165 ± 495	1894 ± 257	1140
* Morelet’s crocodile*	–	2546 ± 78	2339	–	–
* Orinoco crocodile*	3186	2048	4050 ± 314	1870	1657
Q25 (Hz)					
* American alligator*	705 ± 92	435	447 ± 54	393 ± 60	353 ± 28
* Spectacled caiman*	1056 ± 289	618 ± 38	478	–	–
* Nile crocodile*	1615 ± 530	1707 ± 573	1523 ± 350	1609 ± 219	920
* Morelet’s crocodile*	–	1826 ± 46	1741	–	–
* Orinoco crocodile*	2455	1656	1767 ± 251	1457	1491
Q75 (Hz)					
* American alligator*	1377 ± 165	980	1172 ± 232	960 ± 168	934 ± 160
* Spectacled caiman*	2277 ± 493	1804 ± 152	1080	–	–
* Nile crocodile*	3317 ± 761	3115 ± 666	2728 ± 496	2714 ± 252	2110
* Morelet’s crocodile*	–	2982 ± 161	2917	–	–
* Orinoco crocodile*	3484	2369	2737 ± 311	2196	2543
IQR (Hz)					
* American alligator*	672 ± 168	546	727 ± 198	567 ± 127	582 ± 139
* Spectacled caiman*	1221 ± 293	1186 ± 165	601	–	–
* Nile crocodile*	1701 ± 484	1408 ± 279	1204 ± 233	1105 ± 310	1191
* Morelet’s crocodile*	–	1156 ± 128	1176	–	–
* Orinoco crocodile*	1029	713	971 ± 216	739	1052
cent (Hz)					
* American alligator*	1164 ± 106	958	1035 ± 120	884 ± 108	863
* Spectacled caiman*	1766 ± 330	1377 ± 69	1009	–	–
* Nile crocodile*	2490 ± 540	2448 ± 519	2187 ± 338	2161 ± 159	1555
* Morelet’s crocodile*	–	2429 ± 102	2366	–	–
* Orinoco crocodile*	2897	2084	2269 ± 232	1988	2075
skewness					
* American alligator*	2.03 ± 0.07	2.52	2.29 ± 0.48	2.56 ± 0.45	2.87 ± 0.57
* Spectacled caiman*	1.63 ± 0.45	2.21 ± 0.13	2.43	–	–
* Nile crocodile*	1.40 ± 0.34	1.58 ± 0.50	2.05 ± 0.48	2.52 ± 0.36	2.37
* Morelet’s crocodile*	–	1.47 ± 0.19	1.67	–	–
* Orinoco crocodile*	2.20	2.63	2.19 ± 0.62	2.18	3.03
kurtosis					
* American alligator*	6.81 ± 2.25	8.50	7.88 ± 2.72	9.22 ± 2.52	11.30 ± 4.48
* Spectacled caiman*	5.75 ± 2.00	7.80 ± 0.64	8.35	–	–
* Nile crocodile*	4.87 ± 1.41	5.56 ± 2.19	7.69 ± 2.43	10.24 ± 2.08	9.62
* Morelet’s crocodile*	–	4.80 ± 0.69	5.66	–	–
* Orinoco crocodile*	8.62	10.63	8.21 ± 3.92	7.57	12.6
sfm					
* American alligator*	0.385 ± 0.074	0.401	0.430 ± 0.070	0.345 ± 0.065	0.355 ± 0.074
* Spectacled caiman*	0.568 ± 0.059	0.553 ± 0.047	0.374	–	–
* Nile crocodile*	0.734 ± 0.072	0.721 ± 0.065	0.691 ± 0.066	0.629 ± 0.065	0.562
* Morelet’s crocodile*	–	0.721 ± 0.055	0.736	–	–
* Orinoco crocodile*	0.600	0.520	0.607 ± 0.068	0.555	0.647
