# Supplementary material for: Size does matter: crocodile mothers react more to the voice of smaller offspring
Source: Sci Rep. 2015 Oct 23;5:15547. doi: 10.1038/srep15547 (PMC4616036; doi:10.1038/srep15547)
Supplement: Supplementary Audio legend [file srep15547-s1.doc]

**Size does matter: crocodile mothers react more to the voice of smaller offspring**

T. Chabert, A. Colin, T. Aubin, V. Shacks, S. L. Bourquin, R.M. Elsey, J. G. Acosta & N. Mathevon

**Supplementary Audio 1.** Experimental calls (corresponding to the calls illustrated in Figure 2). The file contains 5 successive sounds: 1) a call from a 35.5 cm Nile crocodile juvenile (“small” size); 2) a call from a 64.5 cm Nile crocodile juvenile (“large” size); 3) the synthetic signal SYNTsmall; 4) the synthetic signal SYNTlarge; 5) the synthetic signal NOFM.
